# Lung retention and particokinetics of silver and gold nanoparticles in rats following subacute inhalation co-exposure

**DOI:** 10.1186/s12989-021-00397-z

**Published:** 2021-01-21

**Authors:** Jin Kwon Kim, Hoi Pin Kim, Jung Duck Park, Kangho Ahn, Woo Young Kim, Mary Gulumian, Günter Oberdörster, Il Je Yu

**Affiliations:** 1 Department of Mechanical Engineering, Hanyang University, Ansan, South Korea; 2 Aerosol Toxicology Research Center, HCTm CO.,LTD, Icheon, South Korea; 3 College of Medicine, Chung-Ang University, Seoul, South Korea; 4 National Institute for Occupational Health, Johannesburg, South Africa; 5 Haematology and Molecular Medicine, University of the Witwatersrand, Johannesburg, South Africa; 6 Water Research Group, Unit for Environmental Sciences and Management, North West University, Private Bag X6001, Potchefstroom, 2520 South Africa; 7 Department of Environmental Medicine, University of Rochester, Rochester, NY USA; 8 HCT CO., LTD, Seoicheon-ro 578 beon-gil, Majang-myeon, Icheon, 17383 South Korea

**Keywords:** Gold nanoparticles, Silver nanoparticles, Subacute inhalation exposure, Co-exposure, Particokinetics, Toxicokinetics, Lung retention

## Abstract

**Background:**

Inhalation exposure to nanomaterials in workplaces can include a mixture of multiple nanoparticles. Such ambient nanoparticles can be of high dissolution or low dissolution in vivo and we wished to determine whether co-exposure to particles with different dissolution rates affects their biokinetics.

**Methods and Results:**

Rats were exposed to biosoluble silver nanoparticles (AgNPs, 10.86 nm) and to biopersistent gold nanoparticles (AuNPs, 10.82 nm) for 28 days (6-h/day, 5-days/week for 4 weeks) either with separate NP inhalation exposures or with combined co-exposure. The separate NPs mass concentrations estimated by the differential mobility analyzer system (DMAS) were determined to be 17.68 ± 1.69 μg/m^3^ for AuNP and 10.12 ± 0.71 μg/m^3^ for AgNP. In addition, mass concentrations analyzed by atomic absorption spectrometer (AAS) via filter sampling were for AuNP 19.34 ± 2.55 μg/m^3^ and AgNP 17.38 ± 1.88 μg/m^3^ for separate exposure and AuNP 8.20 ± 1.05 μg/m^3^ and AgNP 8.99 ± 1.77 μg/m^3^ for co-exposure. Lung retention and clearance were determined on day 1 (6-h) of exposure (E-1) and on post-exposure days 1, 7, and 28 (PEO-1, PEO-7, and PEO-28, respectively). While the AgNP and AuNP deposition rates were determined to be similar due to the similarity of NP size of both aerosols, the retention half-times and clearance rates differed due to the difference in dissolution rates. Thus, when comparing the lung burdens following separate exposures, the AgNP retention was 10 times less than the AuNP retention at 6-h (E-1), and 69, 89, and 121 times lower less than the AuNP retention at PEO-1, PEO-7, and PEO-28, respectively. In the case of AuNP+AgNP co-exposure, the retained AgNP lung burden was 14 times less than the retained AuNP lung burden at E-1, and 26, 43, and 55 times less than the retained AuNP lung burden at PEO-1, PEO-7, and PEO-28, respectively. The retention of AuNP was not affected by the presence of AgNP, but AgNP retention was influenced in the presence of AuNP starting at 24 h after the first day of post day of exposure. The clearance of AgNPs of the separate exposure showed 2 phases; fast (T_1/2_ 3.1 days) and slow (T_1/2_ 48.5 days), while the clearance of AuNPs only showed one phase (T_1/2_ .81.5 days). For the co-exposure of AuNPs+AgNPs, the clearance of AgNPs also showed 2 phases; fast (T_1/2_ 2.2 days) and slow (T_1/2_ 28.4 days), while the clearance of AuNPs consistently showed one phase (T_1/2_ 54.2 days). The percentage of Ag lung burden in the fast and slow clearing lung compartment was different between separate and combined exposure. For the combined exposure, the slow and fast compartments were each 50% of the lung burden. For the single exposure, 1/3 of the lung burden was cleared by the fast rate and 2/3 of the lung burden by the slow rate.

**Conclusions:**

The clearance of AgNPs follows a two- phase model of fast and slow dissolution rates while the clearance of AuNPs could be described by a one- phase model with a longer half-time. The co-exposure of AuNPs+AgNPs showed that the clearance of AgNPs was altered by the presence of AuNPs perhaps due to some interaction between AgNP and AuNP affecting dissolution and/or mechanical clearance of AgNP in vivo.

**Supplementary Information:**

The online version contains supplementary material available at 10.1186/s12989-021-00397-z.

## Background

Nanomaterials have many applications. For example, silver nanoparticles are widely used as an antibacterial component in textiles, personal care products, cosmetics, home furnishing appliances, and biomedicine [1, 2], while gold nanoparticles are often used in biomedical fields, such as drug delivery, image diagnostics, and therapies [3–5]. Inhaled airborne particles, such as dust, ultrafine particles, fumes, from environmental and occupational sources are deposited in all regions of the respiratory tract depending on their size [6, 7]. Workers in occupational settings and consumers of nanomaterial-containing products are likely exposed to multiple nanomaterials, including both soluble and poorly soluble nanomaterials. AgNP and AuNP can be representative nanomaterials for soluble and insoluble nanomaterials. Toxicokinetics or particokinetics of nanomaterials including studies on the absorption, distribution, metabolism, and elimination (ADME) of naomaterials are essential in assessing their potential health effects. Recognizing the difference in toxicokinetic evaluation between conventional chemicals including pharmaceuticals and nanomaterials, the current OECD toxicokinetic test guideline 417 explicitly stated that the guideline is not intended for testing nanomaterials [8]. While it is under revision, the newly revised OECD inhalation test guidelines included some portion of toxicokinetics such as lung burden measurement of particulate aerosols including nano-range aerosols. The recently revised OECD guidelines for subacute (TG 412) and subchronic (TG 413) inhalation toxicology testing stated that “testing of poorly soluble solid aerosols should include measurements of lung burden and clearance kinetics” [9, 10]. Therefore, such test guidelines require additional post-exposure observation (PEO) periods that include lung burden measurements to inform on lung clearance behavior and translocation. The guideline and GD (guidance document) 39 recommended 2–3 time points during the post-exposure observation (PEO) to study lung burden after nanoparticle inhalation exposure [9–11]. In a previous toxicokinetic study, the current authors investigated the distribution (particulate or ionic) of AgNPs and AuNPs administered by intravenous injection separately or in combination over 4 weeks, where the nanoparticle clearance was then evaluated during a 4-week recovery period. The results indicated that the AgNPs and AuNPs were distributed in different target tissues depending on their bio-solubility, and that co-administration lowered target tissue levels, suggesting a competitive cellular uptake and confirming that the NP tissue translocation was in a particulate rather than ionic form [12]. The present subacute AgNP inhalation study was also based on the revised OECD test guideline 412. The result suggested that Ag from AgNPs is cleared through two different phases, involving fast and slow clearance. The fast clearance component - which was concentration-dependent - could be related to the rapid dissolution of AgNPs and the slow clearance could be due to mechanical AgNP clearance and low dissolution of AgNPs to form secondary particles originating from silver ions reacting with biogenic anions. These secondary AgNPs might be cleared by mechanisms other than dissolution such as mucociliary escalation, translocation to the lymphatic system, or other organs [13]. A similar observation was made with 20 nm AgNP 1.5 h inhalation exposure and PEO-28 period [14].

However, understanding the biokinetics of NPs following co-inhalation is essential, given that inhalation is the main mode of exposure for workers and consumers and that in the future more frequently the exposure may be simultaneous to more than one NP.

We decided, therefore, to perform subacute (28-days) separate and combined inhalation exposures of rats to AgNPs and/or AuNPs of similar sizes and at similar concentrations. Following exposure, lung burdens were measured at 1, 7, and 28 days post-exposure to determine the clearance kinetics of high dissolution AgNPs and low dissolution AuNPs, and to assess if any interaction may have occurred between these two types of nanoparticles upon co-exposure.

## Materials and methods

### AgNP and AuNP aerosol generation

The aerosol generator consisted of a small ceramic heater connected to an AC power supply that was housed within a quartz tube furnace. The heater dimensions were 50 × 5 × 1.5 mm, and a surface temperature of about 1500 °C within a local heating area of 5 × 10 mm^2^ was achieved within about 10s. For long-term testing, the source materials (about 160 mg), silver wire (100 mg, 99.99% purity, 0.5 mm diameter, Higgslab Co., Ltd., Korea), and gold wire (70 mg, 99.99% purity, 0.5 mm diameter, Higgslab Co., Ltd., Korea), were positioned in a separate ceramic heater at the highest temperature point. The quartz tube was 70 mm in diameter and 140 mm in length. Clean (dry and filtered) air was used as the carrier gas, and the gas flow maintained at 25.0 L/min (Re = 572, laminar flow regime) using a mass flow controller (MFC, AERA, FC-7810CD-4 V, Japan) [15–18]. In the current study, the exposure system consisted of four nose-only chambers; fresh air control, AgNP exposure, AuNP exposure, and AuNP+AgNP co-exposure (Supplement 1). Each generator used 4–5 Lpm (liters per minute), and the remaining air flows of AgNP, AuNP, and AuNP+AgNP were 25.1 ± 0.10 Lpm, 24.8 ± 0.15, and 24.2 ± 0.1 Lpm (AgNP 11.9 ± 0.12 Lpm / AuNP 12.3 ± 0.11 Lpm), respectively. The total airflow in each chamber was 35 Lpm, controlled by the mass flow controller. The airflow from the generators was divided by a valve controller into the AgNP, AuNP, and AuNP+AgNP exposure chambers (NITC, HCT, Icheon, Korea). The target nanoparticle diameter was 10 nm for each nanoparticle exposure, and the target mass concentrations for the AgNP, AuNP, and AuNP+AgNP exposures were 20 μg/m^3^, 20 μg/m^3^, and 10 μg/m^3^ AgNP+ 10 μg/m^3^ AuNP, respectively.

### Monitoring of inhalation chambers and analysis of AgNPs and AuNPs

In each chamber, the nanoparticle size distribution, including the count median diameter (CMD), geometric standard deviation (GSD), particle number, volume, and predicted surface area, were recorded using a differential mobility analyzer system (DMAS) comprised of a differential mobility analyzer (DMA-20, 4220, range 6–225 nm, HCT Co., Ltd. Korea) and condensation particle counter (CPC, 3775, size range 4 nm- 1 μm, TSI INC., Shoreview, MN). Nanoparticles from 6 to 225 nm were measured using sheath air at 15 L/min and polydispersed aerosol air at 1.5 L/min for the DMAS with a density of 10.49 g/cm^3^ for Ag and 19.32 g/cm^3^ for Au, respectively. In addition, the mass concentrations of AgNP and AuNP were determined chemically by using an atomic absorption spectrophotometer (AAS, Perkin-Elmer 900 T, Waltham, MA, USA) after sampling on a mixed cellulose ester (MCE) filter (size: 37 mm and pore size 0.45 μm, SKC, UK) at a flow rate of 1.0 L/min and digesting the samples on a hot plate (PerkinElmer, Concord, ON, Canada) using nitric acid (Fluka, Lot; BCBM5181V). Two samples collected daily from each chamber were analyzed during the 28-day exposure period.

### Transmission electron microscopy

The AgNPs, AuNPs, and AuNPs+AgNPs were collected on a TEM grid (electron microscope, 200 mesh, Formvar/Carbon, TEDpella, CA) and imaged for morphology using a field emission transmission electron microscope (FE-TEM, JEM2100F, 200 kV, JEOL, Tokyo, Japan). Their chemical composition was analyzed using an energy-dispersive x-ray analyzer (EDS, TM200, Oxford Instruments PLC, Oxfordshire, UK), while the CMD and GSD were obtained after measuring 200 particles for each nanoparticle.

### Animal care and housing conditions

Seventy-six male 6-week-old specific-pathogen-free Sprague-Dawley rats (average body weight 178.53 ± 0.63 g) were purchased from OrientBio (Seongnam, Korea) and acclimated for 1 week before commencing the experiments. Three to four rats were housed in polycarbonate cages during the acclimation and experimental period. The animal room temperature, humidity, and light/dark cycle were 21.40 ± 0.55 °C, 48.67 ± 5.56%, and 12 h, respectively. Filtered water and a rodent diet (BSC, Republic of Korea) were supplied ad libitum. The rats were adapted to the nose-only tubes for a week with daily tube placement for 2 h. The 7-week-old rats weighing 273.63 ± 2.83 g were divided into four groups: fresh air control, AgNP, AuNP, and AuNP+AgNP exposure groups sacrifice, and exposed 6 h/day, 5 days/week for 4 weeks. Each exposure group included 19 animals (4 rats for 1-day (6 h) exposure and 5 rats for 1-day, 7-days, and 28-days post-exposure, respectively). The animals were examined daily on weekdays for any evidence of exposure-related effects, including respiratory, dermal, behavioral, nasal, or genitourinary changes suggestive of irritation. The body weights were evaluated at the time of purchase, at the time of grouping, once a week during the inhalation exposure and post-exposure period, and before necropsy (results are not shown). The rat experiments were approved by the Hanyang University Institutional Animal Care and Use Committee in South Korea (HY-IACUC-2017-0143A).

### Quantitative analysis of lung tissue burden using ICP-MS

Immediately after the 6-h. exposure on day 1 and 1, 7, and 28 days after the 28-day exposure period, rats were sacrificed by anesthetizing via an intraperitoneal injection of pentobarbital (EntobarVR, Hanlim Pharm Co. Ltd., Seoul, Korea) at a dose of 150 mg/kg body weight. The animals in the control group were sacrificed first, and all the dissection instruments were thoroughly washed with 70% ethyl alcohol in between the dissections to avoid NP contamination from one organ to another. After measuring the lung weights, the lungs were fixed with 10% neutral buffer formalin for further processing. The fixed lungs were then digested as described in NIOSH 7302 [19] using a microwave (MARS 230/60, CEM, Matthews, NC) with the following three steps: 1) increase the temperature to 110 °C for 15 min; 2) maintain this temperature for 60 min (1600w); and 3) cool for 15 min. The digestion solution for lung tissue consisted of 2 mL of nitric acid (purity of 69.0%; CAS.no of 7697-37-2, Fluka, Germany), and 3 mL of 1% nitric acid to make a final volume 5 ml. The samples were then analyzed using an inductively coupled plasma mass spectrometer (ICP-MS, PerkinElmer NEXION 300S, Concord, ON, Canada). The ICP-MS analysis was conducted according to NIOSH 8200 [20].

The concentrations of Ag and Au in the lungs were determined by ICP-MS based on standard curves established with un-exposed clean lungs spiked with test NPs sampled from the respective inhalation chambers, where the results from digestion, extraction, and dilution were all performed in duplicates. The quantitative analyses for Ag and Au in the lungs were corrected using the spiked standard curve. The recovery yields of AgNPs and AuNPs were 81–113% and 84–105%, respectively, as shown in Supplement 2. The spiked standard curves ranged from 0.2–5 ng/g of lung tissue for AgNPs and 2–100 ng/g of lung tissue for AuNPs. When analyzing the samples, the dilution factor was 100 times. The digestion recovery of AgNPs and AuNPs in the lung tissue was calculated using Eq. 1.

1 $$ \mathrm{Recovery}\kern0.5em \left(100\%\right)=\mathrm{measured}\kern0.5em \mathrm{concentration}\kern0.5em \left(\mathrm{ng}/\mathrm{g}\right)/\mathrm{spiked}\kern0.5em \mathrm{concentration}\left(\mathrm{ng}/\mathrm{g}\right)\times 100 $$

The samples were all analyzed using a standard calibration curve that ranged from 0.05–0.5 ppb for Ag and 1–10 ppb for Au. After analyzing standard blanks 40 times, the measured LOD and LOQ were 0.086 μg /L and 0.260 μg /L, respectively, for Ag and 0.027 μg /L and 0.082 μg/L, respectively, for Au.

### Retention kinetics

The lung retention kinetics for the AgNPs, AuNPs, and AuNP+AgNP co-exposure were determined based on lung burdens measured on 1-day (6-h) of exposure (E-1) and on post-exposure observation days 1 (PEO-1), 7 (PEO-7), and 28 (PEO-28). Half-times were calculated based on the assumption from previous data and our experimental design that Au had a monoexponential decline and Ag had a two- phase decline. The lung clearance kinetics were calculated by applying a first-order decay model and a two-phase model. The first-order model is described by Eq. 2. The two-phase model or two-exponential time-decay function used computer programming based on Eq. 3, prior to which the retention fractions were converted to logarithmic variables. The retention half-time (T_1/2_) was derived using *λ*_1,_ *λ*_2_, and natural log (2) as shown in Eq. 4.

2 $$ \mathrm{M}\left(\mathrm{t}\right)={P}_1\mathit{\exp}\left(-{\lambda}_1t\right) $$

Where

- M(t); lung burden at time (t)
- P1; fraction of lung burden cleared (1.0 for one-compartment model)
- *λ*_1_; clearance rate per day for one-compartment model

3 $$ \mathrm{M}\left(\mathrm{t}\right)={P}_1\mathit{\exp}\left(-{\lambda}_1t\right)+{P}_2\exp \left(-{\lambda}_2t\right) $$

- M(t); lung burden at time (t)
- P1; fraction of lung burden cleared by fast phase
- *λ*_1_; fast clearance rate per day for two-compartment model
- P2; fraction of lung burden cleared by slow phase
- *λ*_2_; slow clearance rate per day for two-compartment model

4 $$ {T}_{1/2}=\frac{\ln (2)}{\lambda}\approx \frac{0.693}{\lambda } $$

- *λ*_1_ is for fast phase half-time (T½_fast_)
- *λ*_2_ is for slow phase half-time (T½_slow_)

### Statistical analysis

An analysis of variance (ANOVA) test and Dunnett T3 multi-range tests were used with up to two points, where one point compared the single and co-exposure groups, while two points compared each group from PEO-1 to PEO-28. The level of significance was set at *P* < 0.05.

## Results

### Characterization of AgNP and AuNP aerosols in inhalation chambers

**Fig. 1:**
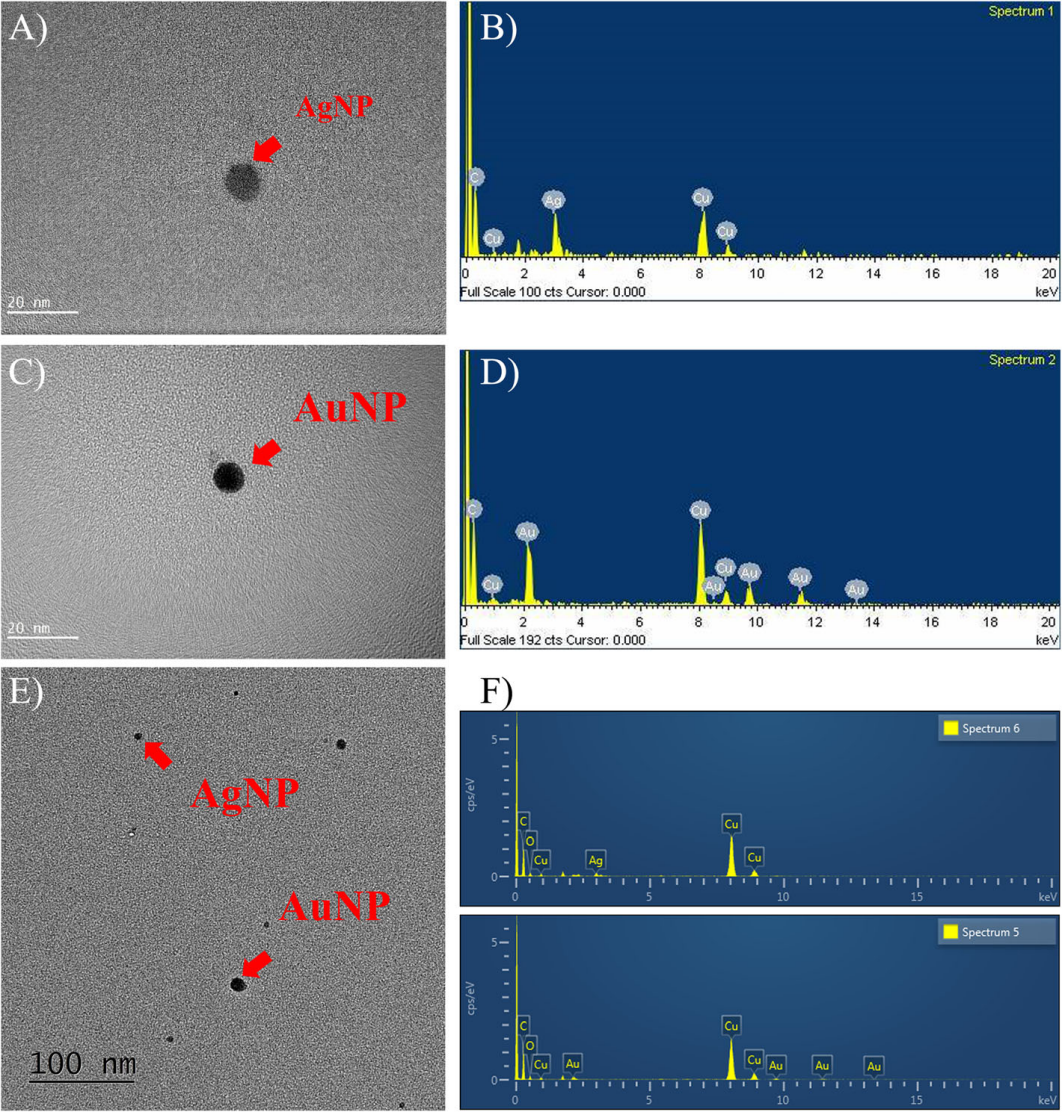
FE-TEM analysis for AgNPs, AuNPs, and AgNP+AuNP co-exposure in chambers; **a** image of single AgNP (scale 20 nm); **b** EDS result for single AgNP; **c** image of single AuNP (scale 20 nm); **d** EDS result for single AuNP; **e** image of AgNP+AuNP co-exposure (scale 100 nm); **f** EDS result for AgNP+AuNP co-exposure

The total number concentrations, CMD, GSD, and surface area of the AgNPs, AuNPs, and AuNPs+AgNPs measured by the DMAS are presented in Table 1 and Supplement 1. FE-TEM revealed non-agglomerated particles (Supplement 3) and TEM-EDS identified AgNP and AuNP particles in each chamber (Fig. 1b and d). The mass concentrations analyzed by AAS via filter sampling were 17.38 ± 1.88 μg/m^3^ for AgNPs, 19.34 ± 2.55 μg/m^3^ for AuNPs for single exposure, and 8.99 ± 1.77 AgNPs + 8.20 ± 1.05 AuNPs for AuNP+AgNP for co-exposure, while the mass concentrations estimated by DMAS were 10.12 ± 0.71 μg/m^3^ for AgNPs and 17.68 ± 1.1.69 μg/m^3^ for AgNPs, respectively. There were some discrepancies in the mass concentrations between filter sampling and DMAS estimation. The discrepancies could be caused by sampling and chemical analysis errors of filter sampling. To avoid these sampling and chemical analytical errors, we used AuNP and AgNP concentrations measured by DMAS rather than AAS based concentrations. Detailed mass calculation from DMAS measurement is described in Supplement 4. TEM indicated that the AgNPs, AuNPs, and AuNPs+AgNPs were the particle diameters log-normally distributed between 6 and 30 nm. The CMD and GSD measurements were 10.40 nm and 1.36, respectively, for the AuNPs, 9.48 nm and 1.49, respectively, for the AgNPs, and 9.00 nm and 1.19, respectively, for the AuNP+AgNP co-exposure (Table 1).

**Table 1:** Aerosol data for AuNPs, AgNPs, and AuNP+AgNP co-exposure

		AuNPs	AgNPs	AuNP+AgNP co-exposure
DMAS^a^	Total particle concentration(#/cm^3^)	1.38 × 10^6^ ± 1.32 × 10^5^	1.44 × 10^6^ ± 1.003 × 10^5^	1.10 × 10^6^ ± 1.110 × 10^5^
	CMD (nm)	10.82 ± 0.24	10.86 ± 0.20	10.91 ± 0.22
	GSD	1.28 ± 0.01	1.27 ± 0.01	1.28 ± 0.01
	Particle mass concentration (μg/m^3^)	17.68 ± 1.69	10.12 ± 0.71	–
	Surface area (nm^2^/cm^3^)	5.79 × 10^8^ ± 7.60 × 10^7^	6.06 × 10^8^ ± 5.591 × 10^7^	5.32 × 10^8^ ± 5.120 × 10^7^
	Volume (nm^3^/cm^3^)	1.28 × 10^9^ ± 2.01 × 10^8^	1.34 × 10^9^ ± 1.358 × 10^8^	1.18 × 10^9^ ± 1.21 × 10^8^
FE-TEM^b^	CMD (GSD)	10.40 (1.36)	9.48 (1.49)	9.00 (1.19)
AAS^c^	Particle mass concentration (μg/m^3^)	19.34 ± 2.55	17.38 ± 1.88	AuNPs: 8.20 ± 1.05 AgNPs: 8.99 ± 1.77

(Mean ± S.D), a) *DMAS* Differential Mobility Analyzing System conducted measurements during 28 days of exposure period; b) CMD and GSD were analyzed based on counts of 200 particles using FE-TEM; c) *AAS* Atomic Absorption Spectrometer; *n* = 40 (2 cases each day for a total of 20 days)

### AgNP and AuNP retention in lungs after 1-day (6-h) of exposure

The whole lung burdens from E-1 and PEO-1 to PEO-28 were analyzed using ICP-MS as described in the Methods. The AgNP and AuNP deposition/retention per lung on day-1 (6-h) of exposure is shown in Table 2 and Supplements 5, 6, 7 and 8 (including lung weights). Despite similar airborne sizes of AgNPs and AuNPs, the retained amount of Ag after 6 h of exposure was much lower (10 times less) than the amount of Au. As discussed later, rapid dissolution and Ag ion clearance may have a significant effect on their retention. Similarly, for the co-exposure of AuNPs+AgNPs, the 6-h retained Ag amount in the lung was also much lower (14 times less) than the amount of Au.

**Table 2:** Lung burden of AuNPs, AgNPs, and AuNP+AgNP co-exposure (ng/lung)

AuNP					
	Single (ng of Au)^A^	Retention %	Co-exposure (ng of Au)^B^	Retention%	B/A
E-1 (4)	466 ± 34	–	313 ± 18	–	0.67 ± 0.04
PEO-1 (5)	8930 ± 742	100.0 ± 0	3607 ± 133^**^	100.0 ± 0.0	0.40 ± 0.01
PEO-7 (5)	8048 ± 1308	90.1 ± 11.6	3137 ± 220^**^	87.0 ± 6.1	0.39 ± 0.03
PEO-28 (5)	7010 ± 578	78.5 ± 6.5	2458 ± 224^a,**^	68.2 ± 6.2	0.35 ± 0.03
AgNP					
	Single (ng of Ag)^C^	Retention%	Co-exposure (ng of Ag)^D^	Retention %	D/C
E-1 (4)	47 ± 5	–	23 ± 1	–	0.49 ± 0.03
PEO-1 (5)	129 ± 15	100.00 ± 0.0	137 ± 6	100.0 ± 0.0	1.06 ± 0.05
PEO-7 (5)	90 ± 8	69.4 ± 5.8	72 ± 9^a^	52.8 ± 6.6	0.80 ± 0.10
PEO-28 (5)	58 ± 11^a^	45.0 ± 8.9	37 ± 5^a,b^	27.4 ± 3.8	0.64 ± 0.09

Unit; mean ± S.E; (), number of animals per group; ng (in whole lung); E1, exposed for 1 day, PEO-1, post-exposure observation day 1; PEO-7 post-exposure observation day 7; PEO-28, post-exposure observation day 28; amount of deposition in whole lung = ng/g lung tissue analyzed by ICP-MS x whole lung weight and it was corrected the lung tissue spiked standard curve; further data details are included in Supplements 5, 6, 7 and 8; the clearance (%) was comparing PEO-1 for each a groups; B/A and D/C represent co-exposure/single exposure; Significant differences using ANOVA of multiple comparison; a) *p* < 0.05, comparing PEO-1; b) *p* < 0.05, comparing PEO-2; **p < 0.05 compared with single and co-exposure

### AgNP and AuNP retention in lungs during post-exposure observation periods

The retained lung burden was expressed as a percent of PEO-1 lung burdens. Thus, at PEO-1, PEO-7, and PEO-28, the retained lung burden of Au was 100 ± 0, 90.1 ± 11.6, and 78.50 ± 6.5%, respectively, for single AuNP exposure. For AuNP+AgNP co-exposure, the retained lung burden of Au at PEO-1, PEO-7, and PEO-28 were 100 ± 0, 87.0 ± 6.1 and 68.2 ± 6.2%, respectively, which is similar as those observed in the single AuNP exposure (Table 2). At PEO-1, PEO-7, and PEO-28, the retained Ag lung retentions were 100 ± 0, 69.4 ± 5.8, and 45.0 ± 8.9%, respectively, for single AgNP exposure. For AuNP+AgNP co-exposure, retained Ag lung burden at PEO-1, PEO-7, and PEO-28 were 100 ± 0, 52.8 ± 6.6 and 27.4 ± 3.8% respectively, which is different from those observed in the single AgNP exposure (Table 2). Since exposure concentrations (μg/m^3^) of AuNP and AgNP in the co-exposure of AuNP + AgNP were nearly half of those of the single exposures, the ratios of retained mass lung burdens on each post-exposure day between single and co-exposures (B/A and C/D in Table 2) was expected to be 0.5 if there was no change in lung retention kinetics. In the case of AuNP single exposure and AuNP+AgNP co-exposure, the ratios of lung burdens of co-exposure/single exposure were < 0.5, ranging from 0.40 ± 0.01, 0.39 ± 0.03, and 0.35 ± 0.03 at PEO-1, PEO-7, and PEO-28, respectively, perhaps indicating either dependence of AuNP clearance on the initial Au lung burden or interference from the presence of AgNPs. In the case of AgNP single exposure and AuNP+AgNP co-exposure, however, the lung burden ratios of co-exposure/single exposure were > 0.5, ranging from 1.06 ± 0.05, 0.80 ± 0.10 and 0.64 ± 0.09 at PEO-1, PEO-7 and PEO-28, respectively, indicating a slower clearance of AgNPs in the presence of AuNPs (Table 2). This ratio for E-1 showed the expected value of 0.49 ± 0.03 for AgNPs indicating that during the initial short 6-h, exposure (E-1) the presence of AuNPs, did not influence AgNP clearance, in contrast to the finding of repeat subacute exposure over 28 days (5 days per week during 4 weeks, total 20 days) showing a lower clearance rate in the presence of AuNPs. The ratio at E-1 for AuNPs was > 0.5, implying also a lower clearance rate in the presence of AgNPs during the short 6-h. exposure; this is opposite to the faster clearance during the subsequent longer exposure over 28 days of 5 days per week for 4 weeks.

### Lung retention kinetics

**Fig. 2:**
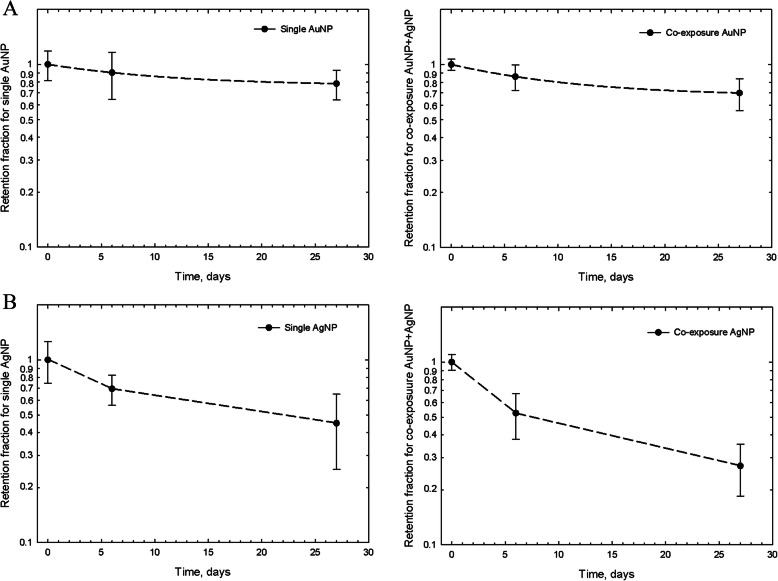
Comparison of AuNPs and AgNPs in lung burden results. **a** Lung burden results of Au. **b** Lung burden results of Ag

Given the limited data points of Au and Ag lung retention in the post-exposure period, possible outcomes consistent with these limited data were calculated for the lung retention kinetics are presented in Table 3 and Fig. 2. Au and Ag lung retention parameters analyzed from the elimination curves are presented in Fig. 2. For Au, 97.9 and 97.1% were retained after 28-day of AuNP and AuNP+AgNP co-exposure, respectively. Calculated retention half times and clearance rates for single AuNP and single AgNP exposure and AuNP + AuNP co-exposure are presented in Table 3. For Single AuNP exposure, a retention half-time (T_1/2_) was 81.5 days, while co-exposure with AgNP reduced the AuNP T_1/2_ to 54.2 days. Given that the retention half times for AuNPs for both scenarios - single and combined exposure - are within the range of normal physiological alveolar clearance rates of 60–90 days for poorly soluble particles in rats [21], a significant difference cannot be substantiated. Therefore, AuNP clearance was likely not influenced by the presence of AgNP co-exposure, which is also supported by the same retained fraction of Au in the lung at day 28 of both single and co-exposure.

**Table 3:** Retention kinetics of AuNPs, AgNPs, and AuNP+AgNP co-exposure in lungs

	First order model	
	T_1/2_, day^a^	
AuNPs	81.5	
AuNPs when co-exposed with AgNPs	54.2	
	Two-phase model	
	Fast-clearance phase	Slow-clearance phase
	T_1/2_, days^a^	T_1/2_, days^a^
AgNPs	3.1	48.5
AgNPs when co-exposed with AuNPs	2.2	28.4

These data were analyzed using a two-phase model (fast and slow); Ag showed a two-phase model, whereas Au showed first order kinetics

The AuNPs and co-exposed AuNPs were analyzed based on a first order model using the equation [M(t)=P_1_exp(−λ_1_t)]

The AgNPs and co-exposed AgNPs were analyzed based on a two-phase clearance (fast and slow) using the equation [M(t)=P1exp(−λ1t)+P2exp(−λ2t)], a) half-time time; T1/2 = ln(2) / λ (d_1_ and d_2_)

In contrast, between 34 and 49% of deposited Ag was estimated to be solubilized and removed from the lung within 5 days after 28-days of AgNP exposure and AuNP-AgNP co-exposure (Fig. 2b). Estimated insoluble AgNPs were retained 66.1 and 51.2% after 28-days of AgNP and AgNP+AuNP co-exposure, respectively. The clearance of single AgNP exposure and AuNP+AgNP co-exposure showed 2 phases for Ag clearance; fast and slow (Fig. 2b). The fast-retention T_1/2_ after single exposure was 3.1 days, and the slow-retention T_1/2_ single exposure was 48.5 days. AuNP+AgNP co-exposure also showed 2 phases of Ag clearance; fast and slow, where the fast-retention T_1/2_ was 2.2 days, and the slow-retention T_1/2_ was 28.4 days. Comparing the clearance of Ag without and with AuNP co-exposure, the slow Ag clearance rate was 1.7-fold faster in the combined exposure group. The clearance of Ag in the co-exposure group was different in the presence of AuNP. However, the percent of lung burden in the fast and slow clearing compartments were also different between single and combined exposure: For the combined exposure, the slow and fast compartment comprised each 50% of the lung burden. For the single exposure, 1/3 of the lung burden was cleared by the fast rate and 2/3 of the lung burden by the slow rate.

## Discussion

This study investigated the differences in the pulmonary deposition/retention and clearance kinetics of AgNPs and AuNPs in rats after daily separate or combined inhalation exposure for 28 days, followed by a 28-day recovery period. Deposition differences due to size or concentration were avoided by using similar concentrations of AgNPs and AuNPs with similar physical and aerodynamic diameters. Thus, similar lung deposition for both NP types was expected, because the observed different retention kinetics between the two NP types following separated and combined exposures must be due to the biosoluble property of AgNP, vs the biopersistent property of AuNPs. It would have been advantageous to have more PEOs for lung tissue sampling and have extended the period beyond PEO-28 because poorly soluble AuNP have a long retention half-time. The rationale for setting three post-exposure time points was based on 1) OECD test guidelines 412 recommends 3 PEOs, 2) OECD GD 39 [11] suggestion “For normal clearance conditions, elimination half-times of particles retained in the lung are in the range of T_1/2_ = 60 to 90 days. Accordingly, post-exposure periods should not be shorter than one generic elimination half-time at normal clearance conditions” 3) Our previous study by Jo et al. [13] indicated AgNP has two phases of elimination; fast (2–4 days) and slow (57–100 days), depending on exposure concentration. Given the well-studied and determined T_1/2_ for inhaled poorly soluble low toxicity particles in rats [22], and the less well-established retention kinetics for biosoluble paritcles, and limited by only 3 post-exposure data points, our experiment was designed to study retention half-times shorter than 60–90 days for AgNP and AuNP. Because we are studying the elimination kinetics of both AgNP and AuNP simultaneously, we set the same three post-exposure time points. Despite the justifications for this decision, three points of PEO to study elimination kinetics are insufficient to establish a comprehensive description of pulmonary Ag and Au retention, and it may only provide estimates. Furthermore, the study design and the values reported in this paper are estimates of these parameters constrained by the sample times in the study design. If more or different time points were collected, the inflection points could be determined more accurately which may result in different pharmacokinetic parameters.

NPs are rapidly removed from the systemic circulation by cells of the mononuclear phagocytic system (MPS) as indicated by the observed distribution of a major fraction of an intravenous injected dose into the spleen and liver, thus equilibrium is not reached. This implies that plasma is usually not a suitable medium to monitor NP exposure and plasma kinetic parameters such as plasma area under the curve (AUC) are generally not relevant [23]. Therefore, sacrificing a large number of animals to obtain tissue distribution data from the PEOs after nanoparticle exposure is required but practically and ethically not possible due to the cost of animal and animal welfare. Best estimates using 3 data points as suggested by the regulatory guideline such as the OECD test guideline. Recently several previously conducted toxicokinetic or biokinetic inhalation studies for nanoparticles including AgNP, AuNP, TiO_2_, and MWCNTs adopted 3 major data points [13, 14, 24–27]. After 5-day inhalation of AuNP (13 nm), 1, 3, and 28-days PEOs showed T_1/2_ of 44.5 days in the lung [24]. Kreyling et al. [25, 26] used 2 hours of nano [^195^Au]AuNP intratracheal inhalation exposure in rats in one study and ^48^V-radiolabeled, 20 nm TiO_2_-NP aerosols in the other study and also chose 1, 7, and 28-days of PEOs to analyze pulmonary retention. They reported short T_1/2_ of 23 days and 25 days, respectively in the lung. These values are shorter than the 60–90 days half-time frequently reported [28, 29]. The authors realized this and reasoned that only three PEO data points of 1, 7, and 28 days are not enough and 28 follow-up is too short to correctly determine the long-term retention half-time. Although we selected the same PEOs in our present study and we agree with this shortcoming, there are clear differences: (i) 2 hours intratracheal inhalation by Kreyling et al. [25, 26] vs 28 days nose-only inhalation in our present study; (ii) rats were anesthetized during intratracheal inhalation vs rats freely breathing during the nose-only inhalation. Although in both scenarios 24 h post-exposure was the first PEO at which point mucociliary deposits, for the most part, have been cleared, it is unknown as to whether anesthesia may have had an initially retarding effect on this fast clearance mechanism. Clearly, the reported T½ of 23 and 25 days [25, 26] are based on a proper mono-exponential modeling of the measured data. Our analogues mono-exponential analysis of the AuNP pulmonary retention (Fig. 2a) resulted in T_1/2_ of 81 (single exposure) and 54 days (co-exposure), the expected range for a poorly soluble low toxicity particle. Our data are estimated from a slope based on only a few data points at the beginning of an exponential process, however, they are consistent with the 60–90 day OECD recommended range, and they are also consistent with results from other longer inhalation studies in rats of poorly soluble particles of low cytotoxicity [30]. Confirmation of this pulmonary T_1/2_ comes from a 28- day rat inhalation study with a poorly soluble low toxicity particle, TiO_2_. Creutzenberg reported the study result of a comprehensive 28-day inhalation – including pulmonary retention kinetic - with a 90-day post-exposure observation time [30]. The study design involved three nano TiO_2_ particle types (rutile, anatase, and the much studied P25, a mixture of both) at 3 concentrations each. Lung retention of the 3 TiO_2_ NPs was similar, the low concentration (3 mg/m^3^) resulting in an average T_1/2_ of 63 days, the mid concentration (12 mg/m^3^) average T_1/2_ of 211 days, high concentration (50 mg/m^3^) average T_1/2_ of 382 days. In addition to confirming the well-established pulmonary T_1/2_ between about 60 and 90 days for low realistic lung burden, this study also confirmed Morrow’s lung particle overload hypothesis of the prolongation of pulmonary particle clearance with excessive lung burdens [22]. Despite the obvious limitations in estimating toxicokinetic parameters and considering also animal welfare, three data points for the lung burden measurement are widely adopted for estmating toxicokinetic patterns for nanoparticles for regulatory requirements.

The completely different retention half-times and clearance rates between these two nanoparticles, where the AuNPs clearance could be expressed by first-order kinetics consistent with alveolar macrophage (AM) mediated mechanical clearance starting 24 h after the last day of exposure when deposits on the tracheobronchial tree had been cleared via the mucociliary escalator. In contrast, the bio-soluble AgNP was eliminated by both mechanical and dissolution clearance. The fast Ag clearance component reflects ionic Ag due to the dissolution of AgNPs, while the slow Ag clearance reflects a combination of mechanical clearance and a possibly lower dissolution of secondary AgNP originating from silver ions reacting with cellular target molecules. Recently published our study [13], lung retention and clearance study after 28-day AgNP exposure of rats with lung burden data on by PEO-1, PEO-7, and PEO-28 also showed a similar result of two different modes of clearance; fast and slow. We suggested that the fast clearance rate, which was concentration-dependent, could be explained by the dissolution of AgNPs and the slow clearance rate was due to slower clearance secondary Ag particles originating from dissolved silver ions after reacting with biogenic anions. These secondary AgNPs might be cleared by mechanisms other than dissolution such as mechanical, translocation along the mucociliary escalator and via the lymphatic system to other organs [13].

AgNPs have been known to undergo diverse biochemical transformations, including accelerated oxidative dissolution in an acidic milieu, thiol binding and exchange, photoreduction of thiol- or protein-bound silver to secondary Ag-NPs, and rapid reactions between silver surfaces and reduced selenium species [31]. Selenide is also observed to rapidly exchange with sulfide in preformed Ag2S solid phases. The combined results allow us to propose a conceptual model for Ag-NP transformation pathways in the human body [31–33]. The formation of secondary AgNPs also occurred in wastewater [34]. These secondary Ag compounds created in the body may be cleared by mechanisms other than dissolution, such as the translocation to the lymphatic system leading to systemic distribution to other organs. In contrast, AuNPs deposited in the lower respiratory tract that is of poor biosolubility – equivalent of a poorly soluble low toxicity (PSLT) particle like TiO_2_ – are mainly cleared at a normal physiological rate after phagocytosis by alveolar macrophages, involving macrophage ingestion, and removal via mucociliary escalator, or translocation to the interstitial lymphatic system. The co-exposure of AuNPs+AgNPs also showed similar results for each NP type, with two-compartment fast and slow phase clearance of AgNPs, although one half of the exposure concentration used with co-exposure resulted in slightly faster clearance rates for both phases than those for single AgNP exposure. The co-exposed clearance of AuNPs also showed a similar trend to single AuNP exposure with a shorter T_1/2_ associated with the lower concentration, although both half-times were still in the range of normal physiological pulmonary particle clearance for rats.

The deposited AgNP can exist in the form of AgNP or Ag ions or secondary Ag compounds, as dissolved forms. The latter may be poorly soluble or insoluble bio-persistent formed by Ag ions reacting with biomolecules forming silver protein complexes in the lung [31, 32]. Non-ionic silver (e.g. metallic) or silver salt is most likely in the form of silver ions after slow or fast dissolution following uptake to the body. Free silver ions are subjected to binding to proteins, peptides, and can become a component of the extracellular matrix (ECM) [33]. When lung tissue bound with components of ECM breaks down, the soluble fragments containing silver ions could translocate to distant locations and deposit, whereas the insoluble fragments containing silver ions could remain in tissue as inert sulphide compounds after endocytosis [31]. Argyrial deposits were found to result from the combined pathways involving partial digestion to soluble silver in the lungs or GI tract, ion uptake, and systemic transport to soluble silver, ion uptake and systemic transport as thiol complexes, photoreduction of Ag (I) (zerovalent Ag) to immobilized silver in the form of AgNP in the near skin region and then in situ transformation to sulfides and selenides [31]. Perhaps, these insoluble silver complexes produced after the fast-phase clearance may be cleared by a slow-phase. Since the fast-phase indicates clearance of dissolved Ag ion, single AgNP exposure and AgNP combined exposure showed the same trend for this faster clearance. The slow-phase clearance may be poorly soluble Ag- or AgNP-complexes which may be influenced by the presence of insoluble AuNP in the co-exposure study. The percentage of lung burden in the fast and slow clearing compartment is different between single and combined exposure. For the single exposure, 1/3 of the Ag lung burden is cleared by the fast rate and 2/3 of the Ag lung burden by the slow rate. In contrast, the slow and fast compartment are each 50% of the Ag lung burden in the co-exposure. A similar observation was reported recently by Kreyling et al. [14] after 1.5 h exposure of ^105^Ag-radiolabeled 20 nm AgNP and thereafter 28-day post-observation. The freshly deposited AgNP first dissolved thereby releasing Ag + ions from their surface. In step 2, a fraction of the ions forms layers of Ag-salt molecules around the AgNP which retards the further release of Ag + ions from the NP surface (step 3). In step 4, the rest of the Ag + ions form Ag-salt molecules of low solubility in the alveolar epithelial lining fluid (ELF) which is rich in Cl^−^, S^− 2^, PO4^− 2^ and Se^− 2^ ions. Due to the high concentration of the Ag-salt molecules, they precipitate to nano-sized clusters (step 5). The Ag-salt clusters scavenge most of the Ag-salt molecules (step 6). Both the cores of AgNP and the Ag-salt clusters are phagocytized by lung surface macrophages (step 7) which will gradually transport them to the distal end of the ciliated airways for mucociliary transport to the larynx where they are swallowed into the gastrointestinal tract (step 8). Alternatively, both particulate species may be endocytosed by cells of the alveolar epithelium (e.g. epithelial type 1 + 2 cells) which may exocytose them in exosomes for translocation across the ABB (air blood barrier (step 9). Translocation across the ABB of both particulate species may also occur directly from the ELF. So insoluble AuNP simultaneously deposited to the alveolar region with AgNP may compete with insoluble components of AgNP in terms of phagocytosis by lung alveolar and interstitial macrophages, endocytosis by epithelial cells, fibroblasts, endothelial cells, etc. Between single exposure of AgNP and combined exposure of AgNP, the observed influence of insoluble AuNP on the clearance of soluble AgNP is not well understood at this time. The underlying differences between AgNPs and AuNPs observed between single and combined exposures require additional investigations, including both in vivo and in vitro studies to highlight the importance of dissolution in cellular retention and clearance.

## Conclusions

This study evaluated the lung retention/clearance and toxicokinetics of AuNPs and AgNPs in rats during a 28-day post-exposure observation period following subacute (28-days) inhalation exposure both separately and combined. The nanoparticle concentrations and diameters were all similar. The clearance of AgNPs was found to have two phases, fast and slow, while the clearance of AuNPs only showed a slow phase. Moreover, in the co-exposure of AuNPs+AgNPs, the clearance AgNPs also showed fast and slow phases, while the clearance of AuNPs showed consistently one slow phase. Moreover, the clearance of AuNPs was not affected by the presence of AgNP co-exposure, but the clearance of AgNP was influenced by the co-exposure of AuNP indicating some unknown interactions in the overall clearance in the presence of poorly soluble and AuNP.

## Supplementary Information

**Additional file 1: Supplement 1.** Schematic of exposure system for generating AuNPs, AgNPs, and AuNP+AgNP co-exposure for nose only exposure chambers.
** Supplement 2.** Spiked standard curve and recovery detection for Au and Ag in lung tissue. Range of Au, 2 – 100 ng/g ; range of Ag, 0.2 – 5 ng/g.
** Supplement 3.** Particle distribution in exposure chambers based on DMAS and FE-TEM.
** Supplement 4.** The particle mass concentration of single of AgNP and AuNP using by DMAS. The particle mass concentrations were calculated based on DMAS data of number concentration and average of diameter and particle density following under formula.
** Supplement 5.** Deposition and retention of Au.
** Supplement 6.** Deposition and retention of Au in case of co-exposure with AgNPs.
** Supplement 7.** Deposition and retention of Ag.
** Supplement 8.** Deposition and retention of Ag in case of co-exposure with AuNPs.

## Data Availability

All data and materials are included in the manuscript, tables, figures and supplements.
