# Supplementary material for: Lung retention and particokinetics of silver and gold nanoparticles in rats following subacute inhalation co-exposure
Source: Part Fibre Toxicol. 2021 Jan 21;18:5. doi: 10.1186/s12989-021-00397-z (PMC7819173; doi:10.1186/s12989-021-00397-z)
Supplement: Supplementary file 1 — Additional file 1: Supplement 1. Schematic of exposure system for generating AuNPs, AgNPs, and AuNP+AgNP co-exposure for nose only exposure chambers. Supplement 2. Spiked standard curve and recovery detection for Au and Ag in lung tissue. Range of Au, 2 – 100 ng/g ; range of Ag, 0.2 – 5 ng/g. Supplement 3. Particle distribution in exposure chambers based on DMAS and FE-TEM. Supplement 4. The particle mass concentration of single of AgNP and AuNP using by DMAS. The particle mass concentrations were calculated based on DMAS data of number concentration and average of diameter and particle density following under formula. Supplement 5. Deposition and retention of Au. Supplement 6. Deposition and retention of Au in case of co-exposure with AgNPs. Supplement 7. Deposition and retention of Ag. Supplement 8. Deposition and retention of Ag in case of co-exposure with AuNPs. [file 12989_2021_397_MOESM1_ESM.docx]

Supplement 1. Schematic of exposure system for generating AuNPs, AgNPs, and AuNP+AgNP co-exposure for nose only exposure chambers.

**
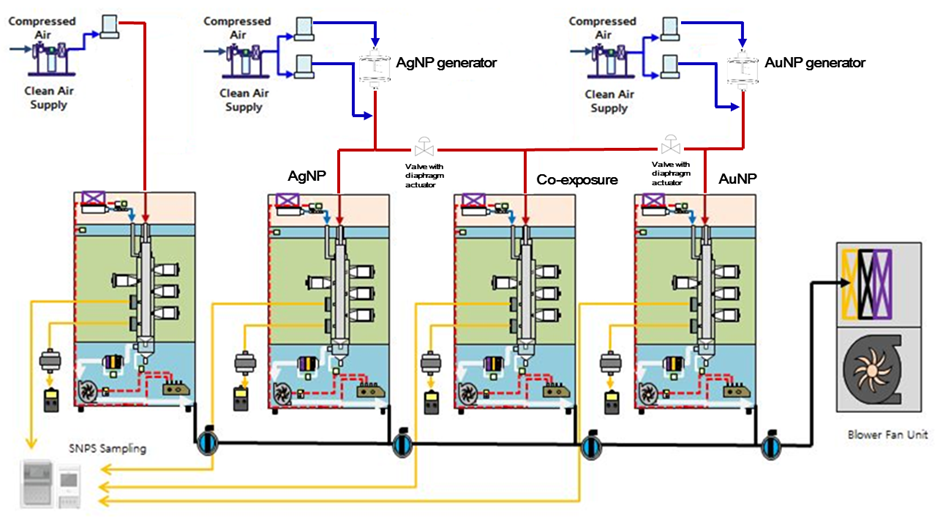
**

Supplement 2. Spiked standard curve and recovery detection for Au and Ag in lung tissue. Range of Au, 2 – 100 ng/g ; range of Ag, 0.2 – 5 ng/g.

Supplement 3. Particle distribution in exposure chambers based on DMAS and FE-TEM; (A), CMD and GSD using DMAS during exposure period; (B), Particle diameter using DMAS; (C), CMD and GSD for AgNPs using FE-TEM; (D), CMD and GSD for AuNPs using FE-TEM; (E) CMD and GSD for AgNP+AuNP co-exposure using FE-TEM.

| (A) CMD and GSD using DMAS during the 28-day exposure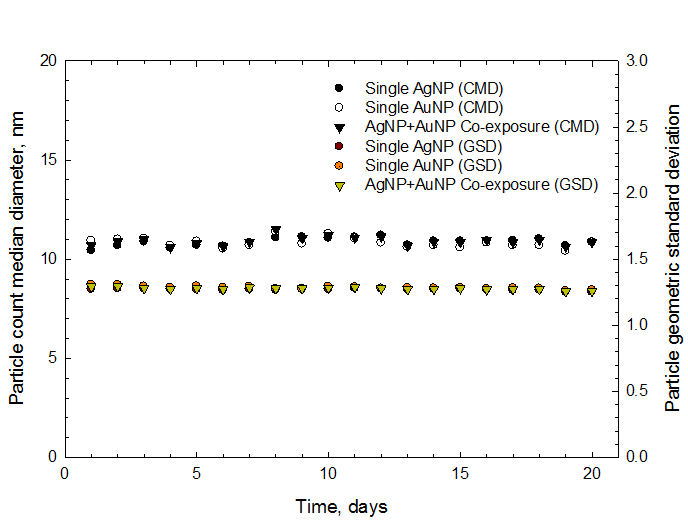 | (B) Size distribution using DMAS  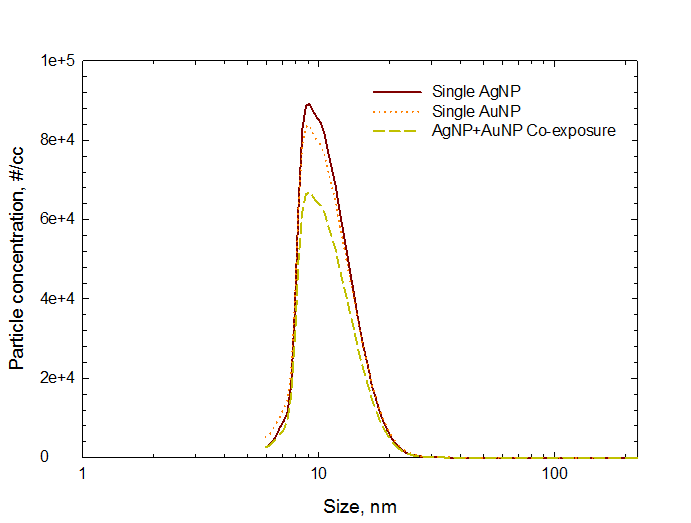 |
| --- | --- |
| (C) Single AgNP using FE-TEM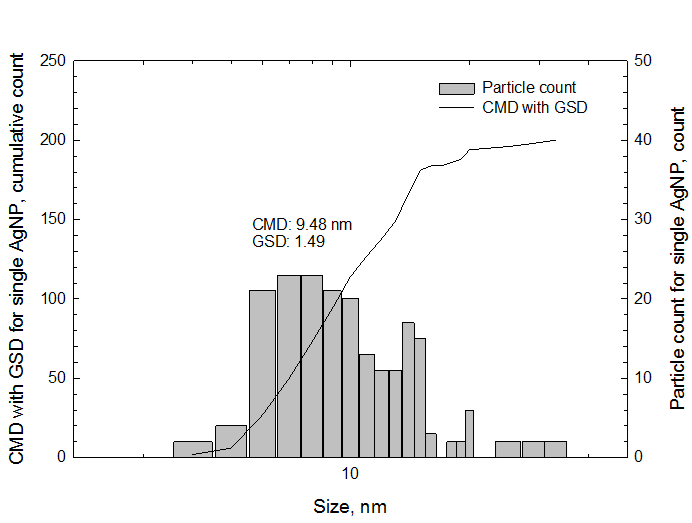 | (D) Single AuNP using FE-TEM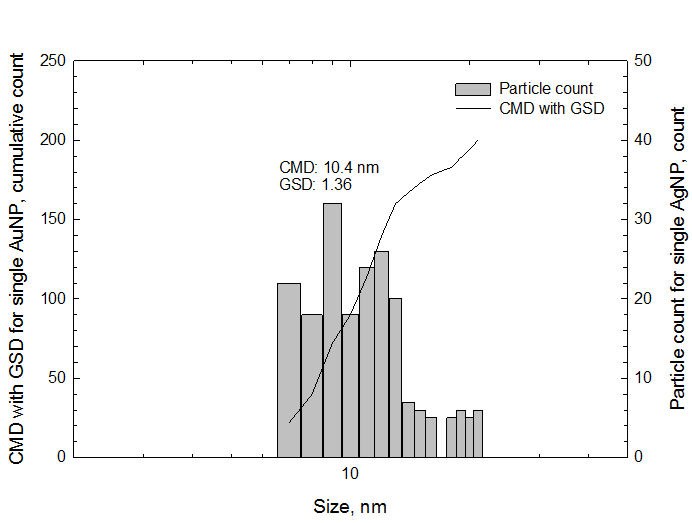 |
| (E) Co-exposure AgNP with AuNP using FE-TEM  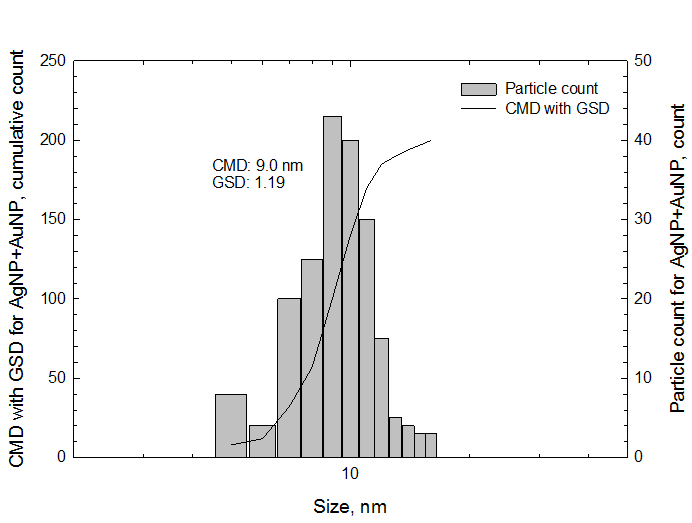 |  |

Supplement 4. The particle mass concentration of single of AgNP and AuNP using by DMAS.

The particle mass concentrations were calculated based on DMAS data of number concentration and average of diameter and particle density following under formula.

A. Mass of one nanoparticle = density x volume of one nanoparticle

- Ag: 10.49 x 10^-12^ ng/nm^3^ x 670.30 nm^3^ = 7.0283 x 10^-9^ ng

- Au: 19.32 x 10^-12^ ng/nm^3^ x 662.92 nm^3^ = 12.80761 x 10^-9^ ng

- Density: 10.49 g/cc of Ag; 19.32 g/cc of Au

- Volume of one nanoparticle: $(\frac{4}{3} \pi r^{3})$

- Average of diameter (based on DMAS): 10.86 nm of Ag (r = 5.43 nm); 10.82 nm of Au (r=5.41 nm)

B. Particle number concentration (based on DMAS)

- Ag: 1.44 x 10^6^ #/cc

- Au: 1.38 x 10^6^ #/cc

C. Particle mass concentration = mass of one particle x particle number concentration

- Ag: 7.0283 x 10^-9^ ng x 1.44 x 10^6^ #/cm^3^ = 10.121 µg/m^3^

- Au: 12.80761 x 10^-9^ ng x 1.38 x 10^6^ #cm^3^ = 17.675 µg/m^3^

Supplement 5. Deposition and retention of Au

|  | µg/g of lung tissue^A^ | Whole lung weight^B^ (g) | µg^C^ (whole lung) |
| --- | --- | --- | --- |
| E-1 | 0.454 ± 0.022 | 1.024 ± 0.032 | 0.466 ± 0.034 |
| PEO-1 | 7.352 ± 0.496 | 1.211 ± 0.029 | 8.930 ± 0.742 |
| PEO-7 | 6.551 ± 0.588 | 1.218 ± 0.070 | 8.048 ± 1.308 |
| PEO-28 | 5.930 ± 0.587 | 1.194 ± 0.052 | 7.010 ± 0.578 |

Supplement 6. Deposition and retention of Au in case of co-exposure with AgNPs

|  | µg/g of lung tissue^A^ | Whole lung weight^B^ (g) | µg^C^ (whole lung) |
| --- | --- | --- | --- |
| E-1 | 0.296 ± 0.010 | 1.055 ± 0.026 | 0.313 ± 0.018 |
| PEO-1 | 3.129 ± 0.060 | 1.153 ± 0.040 | 3.607 ± 0.133 |
| PEO-7 | 2.645 ± 0.185 | 1.189 ± 0.037 | 3.137 ± 0.220 |
| PEO-28 | 2.095 ± 0.180 | 1.173 ± 0.028 | 2.458 ± 0.224 |

Supplement 7. Deposition and retention of Ag

|  | µg/g of lung tissue^A^ | Whole lung weight^B^ (g) | µg^C^ (whole lung) |
| --- | --- | --- | --- |
| E-1 | 0.045 ± 0.001 | 1.055 ± 0.009 | 0.047 ± 0.005 |
| PEO-1 | 0.122 ± 0.014 | 1.065 ± 0.028 | 0.129 ± 0.015 |
| PEO-7 | 0.072 ± 0.007 | 1.261 ± 0.038 | 0.090 ± 0.008 |
| PEO-28 | 0.047 ± 0.010 | 1.260 ± 0.030 | 0.058 ± 0.011 |

Supplement 8. Deposition and retention of Ag in case of co-exposure with AuNPs

|  | µg/g of lung tissue^A^ | Whole lung weight^B^ (g) | µg^C^ (whole lung) |
| --- | --- | --- | --- |
| E-1 | 0.021 ± 0.001 | 1.055 ± 0.026 | 0.023 ± 0.001 |
| PEO-1 | 0.119 ± 0.007 | 1.153 ± 0.040 | 0.137 ± 0.006 |
| PEO-7 | 0.061 ± 0.008 | 1.189 ± 0.037 | 0.072 ± 0.009 |
| PEO-28 | 0.032 ± 0.004 | 1.173 ± 0.028 | 0.037 ± 0.005 |

E1, after 1 day of exposure, PEO-1, after 28 days of exposure; PEO-7, after 7 days of recovery; PEO-28, after 28 days of recovery;

A= ng/g lung tissue analyzed by ICP-MS

B= whole lung weight

C= amount of deposition in whole lung =AxB= ng/g x g = ng
